# Perinatal and early life factors associated with symptoms of depression in Brazilian children

**DOI:** 10.1186/1471-2458-12-605

**Published:** 2012-08-03

**Authors:** Thaís S Pereira, Antônio A Silva, Maria T Alves, Vanda M Simões, Rosângela F Batista, Juliana D Rodriguez, Felipe P Figueiredo, Fernando Lamy-Filho, Marco A Barbieri, Heloisa Bettiol

**Affiliations:** 1 Department of Public Health, Federal University of Maranhão, Rua Barão de Itapary, 155, 65020-070, São Luís, MA, Brazil; 2 Department of Medicine I, Federal University of Maranhão, São Luís, MA, Brazil; 3 Department of Neurosciences and Behavioural Sciences, Faculty of Medicine of Ribeirão Preto, University of São Paulo, Ribeirão Preto, SP, Brazil; 4 Department of Medicine III, Federal University of Maranhão, São Luís, MA, Brazil; 5 Department of Puericulture and Pediatrics, Faculty of Medicine of Ribeirão Preto, University of São Paulo, Ribeirão Preto, SP, Brazil

**Keywords:** Infant, Low birth weight, Depressive symptoms, Child, Premature birth, Social class

## Abstract

**Background:**

Few studies have been conducted on the association between perinatal and early life factors with childhood depression and results are conflicting. Our aim was to estimate the prevalence and perinatal and early life factors associated with symptoms of depression in children aged 7 to 11 years from two Brazilian birth cohorts.

**Methods:**

The study was conducted on 1444 children whose data were collected at birth and at school age, in 1994 and 2004/2005 in Ribeirao Preto, where they were aged 10–11 years and in 1997/98 and 2005/06 in São Luís, where children were aged 7–9 years. Depressive symptoms were investigated with the Child Depression Inventory(CDI), categorized as yes (score ≥ 20) and no (score < 20). Adjusted and non-adjusted prevalence ratios (PR) were estimated by Poisson regression with robust estimation of the standard errors.

**Results:**

The prevalence of depressive symptoms was 3.9% (95%CI = 2.5-5.4) in Ribeirão Preto and 13.7% (95%CI = 11.0-16.4) in São Luís. In the adjusted analysis, in Ribeirão Preto, low birth weight (PR = 3.98; 95%CI = 1.72-9.23), skilled and semi-skilled manual occupation (PR = 5.30; 95%CI = 1.14-24.76) and unskilled manual occupation and unemployment (PR = 6.65; 95%CI = 1.16-38.03) of the household head were risk factors for depressive symptoms. In São Luís, maternal schooling of 0–4 years (PR = 2.39; 95%CI = 1.31-4.34) and of 5 to 8 years (PR = 1.80; 95%CI = 1.08-3.01), and paternal age <20 years (PR = 1.92; 95%CI = 1.02-3.61), were independent risk factors for depressive symptoms.

**Conclusions:**

The prevalence of depressive symptoms was much higher in the less developed city, São Luís, than in the more developed city, Ribeirão Preto, and than those reported in several international studies. Low socioeconomic level was associated with depressive symptoms in both cohorts. Low paternal age was a risk factor for depressive symptoms in the less developed city, São Luís, whereas low birth weight was a risk factor for depressive symptoms in the more developed city, Ribeirão Preto.

## Background

Even though there are no longer any doubts about its occurrence [1], many gaps in knowledge still exist regarding childhood depression. There is no agreement about the criteria to be used to identify this condition in population-based studies. Prevalence varies depending on the age range studied. Factors associated with this disorder are also very different depending on the criterion used to identify depression, the setting and the age range of the studied population [2-4].

The prevalence of depression detected in Brazilian and international studies can vary considerably depending on the age range evaluated (children, adolescents or adults), the instrument for evaluation, cut-off points and the sample selected [3,5-8]. A higher prevalence of depression has been reported among adult females [5,9], among children [10] and adolescents [11] of low socioeconomic condition and among adolescents with low maternal or paternal schooling [12]. However, many questions concerning the risk factors for depression still persist.

Some studies have investigated the association between low birth weight/preterm birth and health problems in later life [13]. There is ample evidence that certain chronic diseases may originate during fetal life. Several studies have suggested that low birth weight may be associated with cardiovascular diseases [14] and diabetes [15]. However, the association between low birth weight/preterm birth and depression has been less intensively studied. Studies have been conducted and their results are conflicting. Some studies have demonstrated an association between low birth weight and depression among children [16], adolescents [7,16,17] and adults [5,6,17-19]. Of these, some have reported a positive association only for adolescent males [18], others only for adolescent [7] and adult females [5,17] and still others for adults of both sexes [6,19]. There are also studies in children [20] and adults [21-23] that did not observe this association. Few studies assessing the association between preterm birth and depression in adolescents [24] and adults [5,6,22] are available, only one of which demonstrated a positive association [24].

Children are a part of the population whose mental health problems are less studied than those of adults. Knowing the risk factors for depression during childhood is essential for a better understanding of the etiology of this disorder and for planning prevention strategies.

The objective of the present study was to estimate the prevalence of depressive symptoms and to assess the association of perinatal and early life risk factors with depressive symptoms in children aged 7 to 11 years belonging to two Brazilian birth cohorts. We hypothesized that low birth weight, preterm birth, low socioeconomic status, low parental age and female sex are associated with a higher risk of depressive symptoms in childhood.

## Methods

### Study sites

The study was based on two birth cohorts, one started in Ribeirão Preto in 1994, and the other in São Luís in 1997/98, and on follow-up studies conducted at school age when the children were 10 to 11 years old in Ribeirão Preto, in 2004/05, and 7 to 9 years old in São Luís, in 2005/06.

Ribeirão Preto is a city located in the Southeast of the country, a wealthy and industrialized region, with a population of 461,427 inhabitants in 1994. Its Municipal-Human Development Index (M-HDI) was 0.855 in 2000[25,26]. São Luís is the capital of the State of Maranhão, with a population of 781,068 inhabitants in 1997. It is located in the Northeast, one of the poorest regions in the country, with an M-HDI of 0.778 in the same year [25,26]. HDI is a composite measure of development that combines indicators of life expectancy, educational attainment and income. It varies from 0 to 1; the higher the value, the more developed the municipality [27].

### Evaluation at birth

A total of 2923 liveborn infants delivered at 10 city hospitals over a period of 4 months (April to August 1994) were included in the first stage of the Ribeirão Preto study, representing 99% of all live births during the period. Stillbirths were not included. Fewer than 5% of births were not interviewed due to early discharge or refusal. Excluding multiple births, the final sample consisted of 2858 births [28].

The first stage of the São Luís study was conducted from March 1997 to February 1998. Systematic sampling was used, stratified according to the number of births in each of the 10 public and private maternities of the city. At each hospital, one in 7 children was systematically selected from a total of 2,542 hospital live births, stillborns and singleton or multiple births of mothers residing in São Luís. The sample was representative of city births since hospital deliveries corresponded to 96.3% of the total. After the exclusion of multiple births (n = 50) and stillborns (n = 48), the final sample consisted of 2,443 births. Losses due to refusals or to the impossibility of locating the mother occurred in 5.8% of the cases [29].

### Follow-up study at school age

Five groups of birth weight were considered for the follow-up study: <1500 g, 1500 to 2499 g, 2500 to < 3000 g, 3000 to <4.250 g, and infants whose birth weight was at least two standard deviations above the mean for the study population, ≥ 4250 g. Infants in the weight range with the lowest number of newborns (<1500 g, 1500 to 2499 g and ≥4250 g) were oversampled in order to increase the study power. Because in the groups <1500 g and ≥4250 g achieved sample size was small for a meaningful analysis, birth weight was then reclassified into low birth weight (LBW <2500 g) and non-low birth weight (NLBW ≥2500g).

A search for the children of the cohort was carried out in the schools of both cities. All parents or persons responsible for the children in the <1500 g, 1500 to 2499 g and ≥4250 g groups and a fraction of one to three of those responsible for children in the groups of normal birth weight were invited by telephone or by mail to participate in the study. In Ribeirão Preto, after the exclusion of deaths during the first year of life (n = 47), 1150 children were eligible for follow-up. The follow-up rate was 68.7% (169 LBW and 621 NLBW children), with 790 children aged 10–11 years participating in the study.

In São Luís, after the exclusion of deaths during the first year of life, 2378 children were alive at one year of life and 926 were eligible for follow-up. Thus, 673 children aged 7–9 years were followed up (72.7% of the original target group), 81 of them being LBW and 592 NLBW.

Losses occurred due to the impossibility of locating the children, to migration, to the fact that the children were not enrolled in school, to refusal on the part of the parents or because two schools in São Luís did not allow the research team to contact the children.

### Sample size and power of the study

A sample of 673 children in São Luís and a sample of 790 children in Ribeirão Preto have an 80% power to detect a minimum 5% absolute difference in the prevalence of depressive symptoms, estimated at about 10%, with a 5% probability of type I error.

### Evaluation of depressive symptoms

The Childhood Depression Inventory (CDI) was used to investigate the presence of depressive symptoms. The CDI is a screening test that evaluates the presence and severity of specific depressive symptoms and is based on a self-report inventory elaborated by Kovacs in 1981, consisting of 27 items with 3 reply options, with the child checking the one that best describes how he/she feels. A version with 20 items was adapted and standardized by Gouveia *et al..*[30] for Brazilian schoolchildren. The following values are attributed to each option of the questionnaire: 0 (first option), 1 (second option) and 2 (third option). At the end of the test the values for each option are summed and the presence of depressive symptoms is established according to the sum obtained. The cut-offs proposed vary. High cut-off scores are more appropriate than low ones when it is important to minimize false positives and low scores are preferred to minimize false negatives. When CDI is used as a general population-based screening, Kovacs recommends the raw score of 20 as a cut off [3]. On the other hand, for screening in clinical settings, a lower cut-off is better because the base rate of depression is expected to be higher [8]. As a screening tool, the CDI can serve to identify children who are “at risk” for a depressive disorder and may require further assessment with a more complex test battery. CDI has an excellent reliability, 0.87, as measured by Cronbach’s alpha [8].

The CDI was applied to subsamples of 774 children in Ribeirão Preto and of 670 children in São Luís, evaluated at school age. In the present study we used the original 27-item version of the CDI because the original version of the inventory showed a higher degree of internal consistency when compared to the reduced version of the instrument [31]. The children completed the CDI with the aid of trained psychologists. When necessary, questions were read to the children and those words/questions that were hard to understand were explained to them. Prevalence of depressive symptoms was considered when CDI ≥20 points. The prevalence of depressive symptoms based on CDI ≥ 17 points was also calculated to allow comparisons with other investigations.

### Explanatory variables

Explanatory variables were collected at the time of the child’s birth by applying a standardized questionnaire to the mother. They were chosen based on having been previously reported to be associated with depression in some studies. The following variables were considered: sex, number of household members (1 to 4, 5 or more), maternal and paternal age in completed years at the time of the child’s birth ( <20, 20 to 34, 35 or more), maternal schooling (0 to 4, 5 to 8, 9 or more years of study), paternal schooling (0 to 4, 5 to 8, 9 or more years of study), occupation of the family head (non-manual, skilled and semi-skilled manual, unskilled manual, and unemployed), mother’s job outside the home, maternal smoking during pregnancy (yes, regardless of the number of cigarettes smoked/no), marital status (married, consensual union, no companion), parity (1, 2 to 4, 5 or more children), low birth weight (<2500 g) and preterm birth (<37 weeks of gestational age measured on the basis of the date of the last normal menstrual period reported by the mother) [29]. Birth weight was measured on Filizola digital scales by trained personnel supervised by the research team.

### Statistical analysis

The response variable was depressive symptoms, categorized into yes (CDI ≥ 20) and no (CDI < 20). Uni- and multivariable analyses were used and the prevalence ratios (PR) and their respective 95% confidence intervals were estimated. Statistical analysis was performed using Poisson regression, with robust adjustment of variance [32]. Multivariable analysis included the variables that showed p < 0.10 and other variables reported to be associated with depression in the literature even though they were not significant in univariable analysis. In multivariable analysis, the level of significance was set at 0.05. Thus low birth weight, preterm birth, parity, maternal schooling, occupation of family head, number of household members, marital status, and maternal and paternal age were included in the multivariable analysis. Models treating birth weight and gestational age as continuous variables including a quadratic term for both were also fitted to explore possible non-linear associations with symptoms of depression. In case non-linear associations were significant birth weight or gestational age were plotted against predicted prevalence of depressive symptoms. Curves were fitted using lowess regression.

Due to the complex sampling design, the sample was not equiprobabilistic, i.e., low birth weight (<2500 g) and high birth weight (≥ 4250 g) children were over-represented in the sample. For this reason, the estimates of prevalence and their respective standard errors were weighted using the set of *svy* commands of the Stata software. The different probabilities of selection of each birth weight group and preterm birth group were taken into consideration in the weighting procedure. Sample stratification according to birth weight was also taken into consideration.

### Ethical Aspects

The reasons for the study and the methodological procedures involved were explained to the parents and/or persons responsible who, after accepting the participation of their children in the study, signed an informed consent form at birth and at follow-up according to the directives and the regulatory norms of research involving human beings of the Brazilian National Health Council, resolution 196/1996 and complementary ones. The right to interrupt the study at any time, access to the results and confidentiality about them were guaranteed to the participants. The project was approved by the Research Ethics Committee of the University Hospital, Faculty of Medicine of Ribeirão Preto, University of São Paulo (HCFMPR-USP, N^o^ 2165/2004) and by the Research Ethics Committee of the University Hospital, Federal University of Maranhão, UFMA (N^o^ 060/2005).

## Results

Children from the Ribeirão Preto birht cohort were a bit older (10–11 years of age) and with higher family income (45.5% earned ≥4 minimum wages) whereas children from the São Luís cohort were younger (7–9 years of age) and presented a lower family income (only 7.3% earned ≥4 mininum wages). White children represented 56.3 of the sample in Ribeirão Preto and 23.5% in São Luís. Birth weight was lower in the Ribeirão Preto (mean = 3030 g, standard deviation = 691 g) than in the São Luís cohort (mean = 3158 g, standard deviation = 558 g). Gestational age was also lower in the Ribeirão Preto (mean = 37.9 weeks, standard deviation = 2.6 weeks) than in the São Luís cohort (mean = 38.7 weeks, standard deviation = 2.2 weeks).

The prevalence of depressive symptoms using the cut-off point of 20 was 3.9% (95%CI = 2.5-5.4) in Ribeirão Preto and 13.7% (95%CI = 11.0-16.4) in São Luís. Using 17 as the cut-off point, the prevalence of depressive symptoms was 5.8% (95%CI = 4.0-7.4) in Ribeirão Preto and 21.9% (95%CI = 18.6-25.2) in São Luís. These differences between cities were statistically significant (P < 0.001).

In univariable analysis, in São Luís, maternal schooling ≤4 years (PR = 2.05) and from 5 to 8 years (PR = 1.74) were risk factors for the presence of depressive symptoms. Sex, low birth weight, preterm birth, number of household members, parity, maternal smoking during pregnancy, maternal and paternal age, mother’s job, occupation of household head, marital status and intrauterine growth restriction were not associated with the presence of depressive symptoms (Table 1).

**Table 1:** Univariable analysis of risk factors for depressive symptoms in children aged 7 to 9 years, São Luís (2005/06)

**Variables**	N*	**% depressive symptoms (weighted)**	**Prevalence Ratio**	**95 % Confidence Interval**
**Sex**				
Male	346	15.1	Reference	
Female	324	12.2	0.80	0.54 - 1.20
**Low birth weight**				
No	590	13.7	Reference	
Yes	80	13.0	0.95	0.49 - 1.85
**Preterm birth**				
No	583	13.3	Reference	
Yes	87	17.6	1.33	0.74 - 2.38
**Maternal schooling (years)**				
0 to 4	102	18.7	2.05	1.15 - 3.65
5 to 8	300	15.9	1.74	1.08 - 2.82
≥9	268	9.1	Reference	
**Number of household members**				
1 to 4	254	14.3	1.07	0.71 - 1.60
≥ 5	415	13.4	Reference	
**Parity**				
1	315	12.4	0.81	0.54 - 1.22
2 to 4	320	15.2	Reference	
≥5	35	11.0	0.72	0.24 - 2.16
Maternal smoking				
No	642	13.6	Reference	
Yes	28	16.9	1.25	0.50 - 3.11
**Maternal age (years)**				
<20	199	16.1	1.30	0.85 - 1.98
20 to 34	440	12.4	Reference	
≥35	31	16.7	1.34	0.56 – 3.24
**Paternal age (years)**				
<20	57	21.9	1.68	0.94 - 2.98
20 to 34	491	13.1	Reference	
≥35	106	12.6	0.97	0.54 - 1.73
**Maternal job**				
No	531	14.9	Reference	
Yes	139	9.5	0.64	0.36 - 1.12
**Occupation of family head**				
Non-manual	105	8.5	Reference	
Manual skilled and semi-skilled	317	14.9	1.75	0.85 - 3.59
Manual unskilled and unemployed	225	15.3	1.79	0.86 - 3.75
**Marital status**				
Married	198	13.2	0.92	0.57 - 1.48
Consensual union	313	14.3	Reference	
No companion	159	13.1	0.92	0.56 - 1.51

*Totals (n = 670) may differ for some variables because of missing data.

In univariable analysis, in Ribeirão Preto, 1 to 4 household members (PR = 0.44) and marriage (PR = 0.37) were protective factors against depressive symptoms. Conversely, maternal schooling ≤4 years (PR = 4.60), low birth weight (PR = 2.72), skilled and semi-skilled manual occupation (PR = 8.67), unskilled manual occupation and unemployment of the family head (PR = 12.57) were risk factors for the presence of depressive symptoms. Sex, preterm birth, parity, maternal smoking during pregnancy, maternal and paternal age, and mother’s job were not associated with depressive symptoms (Table 2).

**Table 2:** Univariable analysis of risk factors for depressive symptoms in children aged 10 to 11 years, Ribeirão Preto (2004/05)

**Variables**	N*	**% depressive symptoms (weighted)**	**Prevalence Ratio**	**95 % Confidence Interval**
**Sex**				
Male	393	4.5	Reference	
Female	381	3.3	0.74	0.35 - 1.55
**Low birth weight**				
No	613	3.5	Reference	
Yes	161	9.5	2.72	1.40 - 5.28
Preterm birth				
No	**595**	**3.9**	Reference	
Yes	179	4.0	1.02	0.47 - 2.21
**Maternal schooling (years)**				
0 to 4	153	7.3	4.60	1.51 - 13.99
5 to 8	296	4.6	2.85	0.96 - 8.49
≥9	261	1.6	Reference	
**Number of household members**				
1 to 4	503	3.0	0.44	0.21 - 0.92
**≥ 5**	**237**	**6.7**	Reference	
**Parity**				
1	306	2.2	0.48	0.19 - 1.23
2 to 4	414	4.6	Reference	
≥5	46	10.0	2.15	0.73 - 6.28
**Maternal smoking**				
No	584	3.6	Reference	
Yes	157	6.2	1.72	0.78 - 3.82
**Maternal age (years)**				
<20	126	3.9	1.01	0.36 - 2.79
20 to 34	554	3.9	Reference	
≥35	93	4.4	1.15	0.40 - 3.29
**Paternal age (years)**				
<20	43	6.7	2.33	0.63 - 8.69
**20 to 34**	**514**	**2.9**	Reference	
≥35	170	5.2	1.78	0.75 - 4.23
**Maternal job**				
No	457	5.2	Reference	
Yes	285	2.4	0.46	0.19 - 1.10
**Occupation of family head**				
Non-manual	151	0.5	Reference	
Manual skilled and semi-skilled	455	4.6	8.67	1.97 - 38.06
Manual unskilled and unemployed	126	6.7	12.57	2.62 - 60.37
**Marital status**				
Married	482	2.7	0.37	0.16 - 0.85
**Consensual union**	**153**	**7.3**	Reference	
No companion	104	6.4	0.88	0.32 - 2.41

*Totals (n = 774) may differ for some variables because of missing data.

In multivariable analysis, in São Luís, maternal schooling ≤4 years (PR = 2.39) and of 5 to 8 years (PR = 1.80) continued to be risk factors for the presence of depressive symptoms. Paternal age <20 years, which was not associated with depressive symptoms in the unadjusted analysis, was an independent risk factor after adjustment (PR = 1.92). Even after adjusting for paternal schooling the association remained significant (data not shown). In Ribeirão Preto, low birth weight (PR = 3.98), manual skilled and semi-skilled occupation (PR = 5.30), manual unskilled occupation and unemployment (PR = 6.65) of the family head continued to be risk factors for the presence of depressive symptoms (Table 3).

**Table 3:** Multivariable analysis of risk factors for depressive symptoms in children aged 7 to 9 years, São Luís (2005/06) and in children aged 10 to 11 years, Ribeirão Preto (2004/05)

**Variables**	São Luís *		Ribeirão Preto *	
	**Prevalence Ratio**	**95 % Confidence Interval**	**Prevalence Ratio**	**95 % Confidence Interval**
**Low birth weight**				
No	Reference		Reference	
Yes	0.98	0.50 - 1.90	3.98	1.72 - 9.23
Preterm birth				
No	Reference		Reference	
Yes	1.24	0.66 - 2.31	0.51	0.18 - 1.43
**Parity**				
1	0.87	0.55 - 1.38	0.52	0.18 - 1.56
2 to 4	Reference		Reference	
≥5	0.48	0.16 - 1.45	1.04	0.30 - 3.56
**Maternal schooling (years)**				
0 to 4	2.39	1.31 - 4.34	0.94	0.27 - 3.22
5 to 8	1.80	1.08 - 3.01	1.24	0.43 - 3.59
≥9	Reference		Reference	
**Occupation of family head**				
Non-manual	Reference		Reference	
Manual skilled and semi-skilled	1.75	0.81 - 3.78	5.30	1.14 - 24.76
Manual unskilled and unemployed	1.64	0.74 - 3.61	6.65	1.16 - 38.03
**Marital status**				
Married	1.22	0.75 - 1.96	0.44	0.17 - 1.14
Consensual union	Reference		Reference	
No companion	0.85	0.49 - 1.45	1.10	0.35 - 3.46
**Number of household members**				
1 to 4	1.00	0.66 - 1.52	0.61	0.26 - 1.44
≥ 5	Reference		Reference	
**Maternal age (years)**				
**<20**	1.05	0.65 - 1.70	0.73	0.22 - 2.46
20 to 34	Reference		Reference	
**≥35**	1.73	0.74 - 4.03	0.55	0.16 - 1.89
**Paternal age (years)**				
**<20**	1.92	1.02 - 3.61	1.08	0.20 - 5.83
20 to 34	Reference		Reference	
**≥35**	0.87	0.47 - 1.59	1.79	0.74 - 4.35

* Models were adjusted for all variables presented in the table.

**Figure 1:**
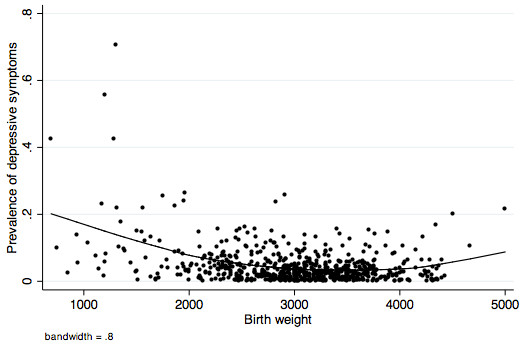
Non-linear association between birth weight and prevalence of depressive symptoms in children aged 10 to 11 years, Ribeirão Preto (2004/05).

In the adjusted models including birth weight and gestational age as continuous variables as well as quadratic terms for both, birth weight (p = 0.018) and its quadratic term (p = 0.037) were associated with depressive symptoms in Ribeirão Preto but not in São Luís. Gestational age and its quadratic term were not associated with symptoms of depression in both cities (data not shown, available on request). In Ribeirão Preto predicted prevalences of depressive symptoms were higher for lower birth weights, decreased from 500 g to 3500 g and increased thereafter (Figure 1).

## Discussion

A high prevalence of depressive symptoms was detected in the present study among children from the less developed city, São Luís. In multivariable analysis in São Luís, lower maternal schooling and young paternal age were independently associated with the presence of depressive symptoms. In Ribeirão Preto, the more developed city, low birth weight and socioeconomic variables (skilled and semi-skilled manual occupation and unskilled occupation and unemployment of the family head) were risk factors for presence of depressive symptoms.

### Prevalence of depressive symptoms

According to the American Academy of Child and Adolescent Psychiatry, the prevalence of depression among children and adolescents aged 9 to 17 years has been estimated at 5% [33]. Using the 27-item CDI, a 11.6% prevalence of possible depression in children aged 11 to 15 years was reported in Northern Ireland using a cut-off point of 17 [34]. In Brazil, using the 20-item CDI and 17 as the cut-off point, prevalences of 1.48% were detected among children aged 7 to 14 years in a private school in Ribeirão Preto [35], of 22% among schoolchildren aged 7 to 17 years in a city in Paraíba, Brazil [36]. Using the 27-item CDI and the same cut-off point, the prevalence of possible depression was 13.7% among schoolchildren aged 7 to 13 years in a school in Minas Gerais, Brazil [37], and of 28.5% among 10- to 17-year-old pupils of a public school in Curitiba, state of Paraná, Brazil [38]. In the present study, using the 27-item CDI and 17 as the cut-off point, the prevalence of depressive symptoms among São Luís children (21.9%) was approximately equal to that detected in Paraíba, Brazil, using the 20-item CDI with the same cut-off point [36]. This prevalence was lower than that detected in another Brazilian study conducted in Minas Gerais that used the 27-item CDI with the same cut-off point [37]. In Ribeirão Preto, the prevalence was much lower, with a value close to those reported in international studies [33,39] and higher than that reported in a study with a similar cut-off point and age range in the same city [35]. In international studies, however, the methods and cut-off points vary considerably [34,40], hampering comparisons.

Several investigations have demonstrated a wide variation in the prevalence of depression in children and differences according to the criteria of diagnostic classification adopted, mainly due to the diverse mode of presentation of depression and the association of this disorder with other psychopathologies. In addition, there still is no single instrument for the evaluation of depression [41]. Among the inventories of self-evaluation of depressive symptoms, the CDI is the one most extensively used and was adapted to Brazil by Gouveia *et al.* in 1995 [30].

Other factors that impair comparison of the results are the different age ranges of the populations studied and the time and location of the studies. More recent studies [38] have reported higher prevalences of depressive symptoms compared to older studies [34]. In addition, studies including older children [38] have reported higher prevalences than studies on younger children [37]. The choice of the sample to be studied can also influence the results since small samples are imprecise and convenience samples, limited to certain public or private schools, are not representative of the target populations [35,37].

The prevalence of depressive symptoms detected in São Luís is similar to that observed by Barbosa and Gaião [36] in Paraíba, a fact that can be explained by the similar socioeconomic and demographic conditions of the two locations. Both São Luís-MA and João Pessoa-PB are located in a less developed region of Brazil, with the highest socioeconomic unequalities in almost all health indicators among children younger than five years. This creates a situation of inequity that makes it difficult for the children to reach their full potential regarding health and physical and mental development [42], possibly explaining the higher prevalences detected in Brazilian studies conducted in less developed regions [36].

### Low birth weight and depressive symptoms

In Ribeirão Preto, the prevalence of depressive symptoms was higher among low birth weight (<2500 g) children compared to normal birth weight children. This result agrees with other studies that showed that LBW, including VLBW (500–1499 g), may influence the development of depression in later life [6,17,18,24]. In multivariable analysis, in Ribeirão Preto, the association between low birth weight and the presence of depressive symptoms remained significant even after adjusting for preterm birth, indicating that intrauterine growth restriction rather than preterm birth might explain this association. Similar results were found in a study in Helsinki [43], where intrauterine growth restriction (IUGR) was also identified as a risk factor for depression.

In São Luís, there was no association between LBW and the presence of depressive symptoms, in agreement with other international studies [20-23]. Mortality in the first year of life was higher in São Luís than in Ribeirão Preto, a fact that may have provoked survival bias and may explain why low birth weight was not associated with depressive symptoms in São Luís. In São Luís, few children with VLBW, which could have been more prone to experiencing depressive symptoms, survived [25]. In addition, oversampling of LBW children was more successful in Ribeirão Preto than in São Luís.

It is known that when children approach adolescence their depressive symptoms tend to resemble those of adults [44]. This may have contributed to explaining why the association between LBW and symptoms of depression was only found in the most developed city, Ribeirão Preto, where children were older.

In Ribeirão Preto, in a model including birth weight, treated as a continuous variable, and a quadratic term for birth weight, association between birth weight and depressive symptoms was non-linear, being higher in the lower birthweights, decreasing from 500 g to 3500 g and increasing thereafter, suggesting that risks of depressive symptoms are higher for both low and high birth weight groups.

Few studies have been published on the association between LBW and depression at school age [7,20,24]. Most studies involved older populations such as young adults [21], middle-aged adults [18], or elderly persons [5,9,17,22,23]. The variety of methods used in previous studies for the evaluation of depression may have been a factor contributing to the conflicting results detected in the literature and in the present study.

### Social factors and depressive symptoms

A worse socioeconomic situation assessed on the basis of maternal schooling and occupation of family head was an important predictor of the presence of depressive symptoms in both cities, in agreement with other studies [5,11,18]. Manual occupation or unemployment of the family head has also been associated with depressive symptoms in some studies [11,18,45]. These findings suggest that disadvantaged social environments may have adverse consequences on mental health because of their effects on psychological development [11]. In multivariable analysis, lower maternal schooling was a risk factor for the presence of depressive symptoms in São Luís. The lower the educational level of the parents, the worse tend to be the physical and emotional conditions for the development and stimulation of the children [10,11,17], since these parents are assumed to have less access to information and therefore interact poorly with their children.

### Young paternal age and depressive symptoms

In our study young paternal age (<20 years) was independently associated with increased depressive symptoms in the less developed city, São Luis [46]. Some young fathers present low level of emotional maturity and lower financial stability, with consequent lower emotional support for their child. This may predispose their children to depression [47]. However, the association between young paternal age and mental health problems has been little researched. In one study low paternal age has been associated with poor mental health, as measured by the Strenghts and Difficulties Questionnaire [48]. Nevertheless, most studies identified advanced but not young paternal age as a risk factor for psychiatric disorders, including schizophrenia [46,49]. In one recent study, young paternal age was associated with increased odds of major depressive disorder, in agreement with our finding in the less developed city [50]. In contrast, in the more developed city we did not find an association between young paternal age and depressive symptoms. It is possible that psychosocial strains associated with young paternal age were buffered in the more developed city. This association suggests that increasing paternal age at pregnancy might reduce depressive symptoms.

### Sex and depressive symptoms

Sex was not associated with the presence of depressive symptoms, in contrast to some international studies, which reported that depression was more prevalent among adolescent or adult females [5,9]. This finding may be explained by the fact that children in our sample were aged 7–11, in contrast to most studies, which included adolescents or adults. It seems that in young children depression does not differ according to sex, whereas as they enter adolescence girls tend to report higher rates of depression than boys [9].

### Strengths and limitations

This was one of the few cohort studies carried out to assess the prevalence of risk factors for depressive symptoms in children in a middle-income country, by comparing a more developed city to a less developed one. The over-representation of the groups of high and low birth weight was a strategy used to increase the statistical power of the study.

The high rates of loss due to mortality, migration and failure to locate many children was a limitation of the study. Participation rates were lower in Ribeirão Preto for mothers who were less than 20 years of age (p = 0.005), who cohabited (p < 0.001) or had ≤ 4 years of full time education (p < 0.001) in comparison to eligible children who did not participate. In São Luís, a lower proportion of children were born to mothers with ≥ 12 years of full time education (p < 0.001), who were primiparous (p = 0.049) or gave birth to males (p = 0.001) among those who participated in the study in comparison to the eligible group who did not participate [25]. These differences may have led to an overestimate of the prevalence of depressive symptoms in São Luís, where the follow-up rate for the group with high schooling was very low.

The CDI is a screening test. Although it can serve as an aid in the diagnostic process, it cannot yield a diagnosis by itself [8]. It could be that the high prevalence of depressive symptoms found in our study, especially in the less developed city, São Luís, was due to the fact that we used a screening instrument. However, the cut-off point 20 used in the present study is recommended when the CDI is used as a general population-based screen test, to minimize false positives [3].

The age difference of two years may have contributed to explaining part of the differences in depressive symptoms between the two cohorts. Children from São Luís were a little younger than those from Ribeirão Preto and may have had difficulties in describing and evaluating their feelings in the last fortnight, as asked by the CDI. A study has shown that the reliability of the child's report of the Diagnostic Interview Schedule for Children (DISC) increased with age and was lower for children aged 6–9 than for those aged 10–13 and 14–18 [51]. However, the prevalence of depression tends to increase with age and thus higher prevalences would be expected in Ribeirão Preto, where children were older. Another limitation was that we only used self-reporting of depressive simptoms and did not assess teacher or parental reports.

## Conclusions

The prevalence of depressive symptoms was much higher in the less developed city, São Luís, than in the more developed one, Ribeirão Preto, and than the values reported in various international studies. Low socioeconomic level was associated with the presence of depressive symptoms in both cohorts. Low birth weight was a risk factor for the presence of depressive symptoms in the more developed city, Ribeirão Preto, only. Young paternal age was associated with a higher report of symptoms of depression in the less developed city, São Luís, only. Improvement of the socioeconomic situation, increasing paternal age at childbirth and the prevention of LBW are measures that could reduce the prevalence of depressive symptoms. However, most of the variation in the prevalence of depressive symptoms was not explained by the variables present in the model.

## Competing interests

The authors declare that they have no competing interests.

## Authors’ contributions

AAS, MAB and HB conceived the study. AAS and TSP performed the statistical analysis. RFB, MTA, AAS, TSP, VMS, FLF, FPF, JDR, MAB and HB contributed to data analysis and interpretation of results. AAS and TSP wrote the paper. All authors read and approved the final version of the manuscript.

## Pre-publication history

The pre-publication history for this paper can be accessed here:

http://www.biomedcentral.com/1471-2458/12/605/prepub
